# Supplementary material for: Effectiveness Evaluation of a Primary School-Based Intervention against Heatwaves in China
Source: Int J Environ Res Public Health. 2022 Feb 22;19(5):2532. doi: 10.3390/ijerph19052532 (PMC8909389; doi:10.3390/ijerph19052532)
Supplement: Supplementary file 1 [file ijerph-19-02532-s001.zip › ijerph-1582552-supplementary.pdf]

Table S1. Implementation frequency of health education activities at intervention school from May to September in 2017

Health education activities	Frequency
Health education curricula, including topic lectures, watching related cartoons, etc.	once every two weeks
Performing a skit or topic class meeting	once
Hand-copied newspapers	once
Drawing contest	once
Theme essay or writing contest	once
Speech contest	once
Recess radio broadcasting	once a week
Blackboard newspapers	two issues
School summer activities: during the school summer holiday, the students were required to participate in the practice activities against extreme heat, such as observing and writing down how the families or friends respond to extreme heat	at least 3 times for every student
Medicine prepare: the school clinic was required to prepare enough medicine to prevent and treat heatstroke in summer.	
Warm boiling water prepare: enough warm boiling water was provided to ensure that students could drink clean warm water at all times.	
Develop habit of washing hands: superintend and help students to develop the habit of washing hands before eating and after using the toilet.	

Table S2. Quantitative scores for knowledge, attitude and practice (KAP) items

Primary students			Parents		
KAP		Score	KAP		Score
	**Knowledge (K, Total score: 8)**	$k_1+k_2+k_3+k_4+k_5/8+K_6/6+K_7/9+K_8/6=8$		**K (Total score: 8 points)**	$k_1+k_2+k_3+k_4+k_5/8+K_6/6+K_7/9+K_8/5=8$
k_1	Climate change	1	k_1	Climate change	1
k_2	Threshold of hot weather	1	k_2	Heat warning	1
k_3	Heatwave	1	k_3	Threshold of hot weather	1
k_4	The grading of heat warning	1	k_4	Heatwave	1
k_5	Impacts of climate change	8	k_5	Impacts of climate change	8
k_6	Climate change mitigation measures	6	k_6	Climate change mitigation measures	6
k_7	Heatstroke symptom	9	k_7	Heatstroke symptom	9
k_8	Heatstroke treatment	6	k_8	Heatstroke treatment	5
	**Attitude (A, Total score: 3)**	$a_1/10+a_2/10+a_3/10=3$		**A (Total score: 3 points)**	$a_1/10+a_2/10+a_3/10=3$
a_1	Willing to learn relevant knowledge	10	a_1	Willing to pay attention to climate change	10
a_2	Willing to start from self	10	a_2	Be interested in relevant knowledge	10
a_3	Willing to change habits	10	a_3	Willing to attend relevant activities	10
	**Practice (P, Total score: 4)**	$p_1+p_2/3+p_3+p_4=4$		**P (Total score: 4 points)**	$p_1+p_2/3+p_3+p_4/7=4$
p_1	Pay attention to weather forecast	1	p_1	Pay attention to weather forecast	1
p_2	Appropriate behavior in hot weather	3	p_2	Use air conditioning	1
p_3	Use air conditioning	1	p_3	Actively consult impacts of weather	1
p_4	Wash hands before meals	1	p_4	Appropriate activities in hot weather	7

Table S3. Difference in age and sex ratio among students participated in different numbers of activities

Number of activity attended	Number of students	Age			Sex			
		Mean±SD (years)	F value	P value	Number of boys	Number of girls	Chi-Square	P value
≤3	224	10.3±1.0			129	95		
4-6	108	10.5±1.0	24.1	<.0001	48	60	5.1	0.0783
7-9	73	11.2±0.7			38	35		

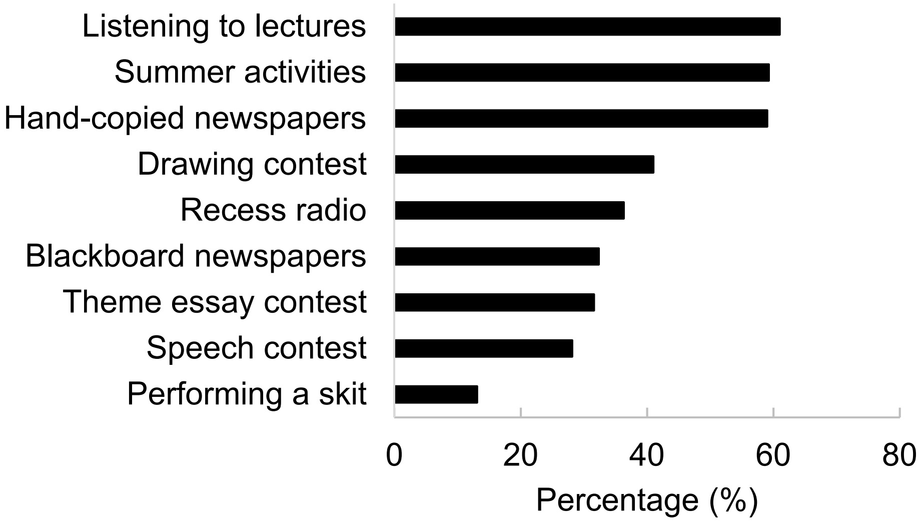

Figure S1. The percentage of primary school students that participated in various health education activities at the intervention school in Dongtai in 2017
